# Association between mental health service utilization and diabetes-hypertension comorbidity: a community-based study

**DOI:** 10.3389/fpubh.2026.1835225

**Published:** 2026-07-03

**Authors:** Penghua Zuo, Xu Yang

**Affiliations:** 1 Melbourne School of Population and Global Health, Faculty of Medicine, Dentistry and Health Sciences, The University of Melbourne, Melbourne, VIC, Australia; 2 School of Public Health, Xinjiang Medical University, Ürümqi, China

**Keywords:** depression, diabetes-hypertension comorbidity, health behaviors, mental health services, propensity score matching

## Abstract

**Objective:**

To examine the association between mental health service (MHS) utilization and diabetes–hypertension (DM–HTN) comorbidity and to explore the roles of depression, health behaviors, and subgroup heterogeneity in this association.

**Methods:**

Data were obtained from a community-based cross-sectional survey of 1,307 adults from 10 communities in Urumqi, China, conducted between July and August 2025. Directed Acyclic Graphs (DAGs) were used to identify confounders, and Propensity Score Matching (PSM) was applied to balance exposure groups. Multivariable logistic regression was used to estimate associations between MHS utilization and DM–HTN comorbidity. Interaction analyses and predicted probability analyses were conducted to assess heterogeneity across population subgroups.

**Results:**

After PSM adjustment, MHS utilization was associated with lower odds of DM–HTN comorbidity (OR = 0.584, 95% CI: 0.360–0.915). Depression (OR = 1.730, 95% CI: 1.024–2.862), smoking (OR = 1.600, 95% CI: 1.177–2.171), and alcohol consumption (OR = 1.720, 95% CI: 1.253–2.350) were positively associated with comorbidity. Compared with no exercise, engaging in 0.5–<1 h (OR = 0.614, 95% CI: 0.379–0.984) and 1–<2 h (OR = 0.355, 95% CI: 0.177–0.671) of exercise per session was associated with lower odds of comorbidity. Interaction analysis identified a significant interaction between MHS utilization and depression status (OR = 0.587, 95% CI: 0.359–0.960), whereas interactions with age, sex, and income were not significant. Predicted probability analysis showed that the absolute reduction in DM–HTN comorbidity associated with MHS utilization was greater among individuals with depression (3.03%) than among those without depression (1.87%).

**Conclusion:**

MHS utilization was associated with a lower likelihood of DM–HTN comorbidity, particularly among individuals with depression. Depression, smoking, alcohol consumption, and insufficient physical activity were associated with increased comorbidity risk. Integrating mental health services with behavioral risk-factor management may contribute to more effective prevention and management of DM–HTN comorbidity.

## Background

Diabetes and hypertension are among the most prevalent non-communicable diseases globally and frequently co-occur as a major form of multimorbidity (1). Population-based studies consistently show strong bidirectional associations between the two conditions, with individuals who have one condition facing substantially increased risk of developing the other (2). The coexistence of diabetes and hypertension greatly elevates the likelihood of cardiovascular, renal, and microvascular complications, resulting in considerable healthcare burden and increased premature mortality (3).

Studies suggest that depression, anxiety, and psychological stress may influence the development of Diabetes-Hypertension (DM-HTN) comorbidity through various mechanisms: (1) Chronic psychological stress can activate the hypothalamic–pituitary–adrenal (HPA) axis, leading to increased cortisol secretion, which in turn induces insulin resistance and hypertension (4); (2) Psychological stress can trigger sympathetic nervous system overactivation, elevating catecholamine levels, increasing blood pressure, accelerating atherosclerosis, and impairing insulin sensitivity (5); (3) Individuals with poor mental health are more likely to adopt unhealthy lifestyles, such as reduced physical activity, poor dietary habits, smoking, excessive alcohol consumption, and sleep disturbances (6), all of which further increase the risk of DM-HTN comorbidity. Furthermore, individuals with depression and anxiety often demonstrate poor disease self-management, with lower adherence to medication, dietary control, and lifestyle interventions, which accelerates disease progression and undermines treatment outcomes (7).

Mental health services (MHS) are increasingly recognized as an important component of chronic disease management for individuals with diabetes and hypertension. MHS encompass psychological counseling, cognitive–behavioral therapy (CBT), pharmacological treatment, and structured psychosocial support, all aiming to improve emotional well-being and reduce physiological stress responses. Evidence suggests that MHS may influence cardiometabolic outcomes through several pathways. First, Mental health services may reduce stress-related autonomic activation, including heightened sympathetic nervous system activity, thereby potentially contributing to improved blood pressure and glycemic regulation (7). Second, engagement with mental health services has been consistently linked to more favorable health-related behaviors, including higher levels of physical activity, healthier dietary and sleep patterns, and lower prevalence of tobacco and alcohol use, likely through improvements in psychological well-being, stress regulation, and self-management capacity (8). Third, by improving mood and coping capacity, mental health services may enhance treatment adherence and self-management behaviors, thereby supporting more effective glycaemic control and overall cardiometabolic management (9).

However, the effectiveness of Mental health services (MHS) in the management of Diabetes-Hypertension (DM-HTN) comorbidity remains controversial. Some cohort studies and randomized controlled trials (RCTs) have reported better blood glucose and blood pressure control and enhanced disease management in patients receiving MHS (10, 11). On the other hand, some studies have found considerable heterogeneity in intervention outcomes, with no significant physiological improvement observed in certain patient groups (9, 11). These inconsistencies may stem from differences in intervention methods, individual adherence, and the conversion effect of psychological improvements into physiological benefits. Therefore, it is imperative to further clarify the specific effects of MHS on DM and HTN comorbidity, explore the underlying mechanisms, and evaluate their practical value in chronic disease management.

Current research in this area also faces methodological limitations. Most studies adopt observational designs, which are susceptible to selection bias and confounding. For example, individuals receiving Mental health services (MHS) may have more severe underlying health conditions, making causal direction difficult to determine (12). Moreover, many studies fail to adequately account for unmeasured confounders such as socioeconomic status and cultural background, potentially affecting the generalizability of findings (13). Although RCTs are considered the gold standard for causal inference, large-scale implementation in MHS research poses challenges due to issues with patient adherence and long-term follow-up feasibility.

To improve the reliability of causal inference, this study employs Directed Acyclic Graphs (DAGs) and Propensity Score Matching (PSM). DAGs help identify potential confounders and reduce bias arising from model misspecification (14). However, constructing a DAG requires domain expertise and prior literature support, which introduces subjectivity and the risk of incorrect assumptions about causal pathways (15). PSM mitigates confounding by matching individuals with similar characteristics, thereby enhancing the robustness of estimates. Nevertheless, PSM cannot fully eliminate unobserved confounding, and the matching process may reduce sample size, affecting statistical power (16). Despite their limitations, the combined use of DAG and PSM can complement each other, enhancing the capacity for valid causal inference. This study utilizes DAGs to identify key confounders and applies PSM to match subjects, thereby more accurately assessing the impact of MHS on DM-HTN comorbidity.

Therefore, this study aimed to examine the association between mental health service utilization and DM-HTN comorbidity among community-dwelling adults in Urumqi, China. DAGs and PSM were applied to reduce observed confounding and improve the robustness of the findings. The findings aim to enrich the theoretical understanding of mental health in chronic disease management and provide empirical evidence to inform public health policies. Ultimately, the study seeks to optimize chronic disease prevention and control strategies and improve the quality of life among affected populations.

## Materials and methods

### Study design and participants

This community-based cross-sectional study was conducted in Urumqi, Xinjiang, China, between July and August 2025. The study aimed to investigate the association between mental health service utilization and diabetes–hypertension (DM–HTN) comorbidity among community residents. Eligible participants were permanent residents aged 18 years or older who had lived in the selected communities for at least 6 months prior to the survey and were able to complete the questionnaire independently or with assistance from trained investigators. Individuals who were unable to communicate effectively due to severe cognitive impairment, serious mental disorders, or other health conditions were excluded. A total of 1,398 questionnaires were distributed, and 1,342 were returned. After data cleaning and quality assessment, 1,307 questionnaires were retained for the final analysis, yielding a valid response rate of 93.49%.

### Sampling procedure

A multistage random sampling strategy was employed to recruit study participants. In the first stage, 10 communities were randomly selected from Urumqi, Xinjiang. In the second stage, approximately 140 permanent residents were randomly selected from each community using simple random sampling. To ensure the quality and consistency of data collection, all investigators received standardized training before the survey. The training covered participant recruitment, questionnaire administration, informed consent procedures, and quality control requirements. During the survey, trained investigators administered the questionnaires using standardized instructions and provided clarification when necessary. Following data collection, all completed questionnaires underwent double-checking and logical consistency verification. Questionnaires with substantial missing information, obvious logical inconsistencies, or invalid responses were excluded from the final analysis.

### Data collection and questionnaire

Data were collected using a structured questionnaire administered through face-to-face interviews conducted by trained investigators. The questionnaire was developed based on previous population health surveys and consisted of four major sections: (1) sociodemographic characteristics, including age, sex, ethnicity, education level, marital status, employment status, and household income; (2) health-related behaviors, including smoking, alcohol consumption, physical activity, and sleep duration; (3) mental health status, assessed using the Patient Health Questionnaire-9 (PHQ-9); and (4) chronic disease history and health service utilization, including physician-diagnosed diabetes, hypertension, and utilization of mental health services. To ensure data quality, investigators received standardized training before the survey. Unified instructions were used during questionnaire administration, and completed questionnaires were reviewed for completeness and logical consistency immediately after collection.

### Definition and measurement of variables

The primary outcome was diabetes–hypertension (DM-HTN) comorbidity. Participants were asked whether they had ever been diagnosed with diabetes or hypertension by a physician or other healthcare professional. Individuals reporting both physician-diagnosed diabetes and physician-diagnosed hypertension were classified as having DM-HTN comorbidity.

The primary exposure variable was mental health service (MHS) utilization. Participants were asked whether they had ever utilized mental health services provided by qualified healthcare professionals or mental health institutions past 12 months, including psychological counseling, psychiatric consultation, psychotherapy, or other mental health-related services. Responses were categorized as “yes” or “no.” Information regarding the frequency and duration of mental health service utilization was not collected.

Depression status was assessed using the validated Patient Health Questionnaire-9 (PHQ-9). The PHQ-9 consists of nine items evaluating depressive symptoms over the previous 2 weeks, with total scores ranging from 0 to 27. A PHQ-9 score of 10 or higher was used to indicate clinically significant depression.

Smoking status was defined as current smoking, including both daily smokers and occasional smokers. Alcohol consumption was defined as having ever consumed any type of alcoholic beverage.

Physical activity was assessed based on moderate- or vigorous-intensity activities. Moderate-intensity activities were defined as activities causing a moderate increase in breathing or heart rate, whereas vigorous-intensity activities were defined as activities causing a substantial increase in breathing or heart rate. Participants reported both exercise frequency and average exercise duration per session.

Sleep behavior was evaluated using self-reported sleep duration on workdays and non-workdays. Participants were asked to report their average daily sleep duration separately for workdays and non-workdays.

Based on the Directed Acyclic Graph (DAG), the minimally sufficient adjustment set included depression status, smoking status, alcohol consumption, exercise frequency, exercise duration, sleep duration on workdays, and sleep duration on non-workdays. These variables were included in the propensity score matching and subsequent regression analyses to reduce potential confounding.

### DAG construction

A Directed Acyclic Graph (DAG) was constructed based on prior literature and domain knowledge to represent the assumed relationships among mental health service utilization, diabetes–hypertension comorbidity, and relevant covariates. The DAG was drawn using the dagitty and ggdag packages in R.

The backdoor criterion was applied to identify confounding variables. Based on prior literature and the conceptual DAG, age, gender, education, income, and marital status were treated as pre-exposure confounders and were included in the propensity score model. Depression, smoking, drinking, physical activity, and sleep-related variables were subsequently incorporated in extended regression models to explore potential behavioral and psychological pathways linking mental health service utilization with DM–HTN comorbidity. These variables were used for propensity score estimation and subsequent adjustment analyses.

### PSM and statistical analysis

Propensity score matching (PSM) was performed to reduce potential confounding between mental health service utilization and diabetes–hypertension comorbidity. Propensity scores were estimated using a logistic regression model that included depression, smoking status, alcohol consumption, exercise frequency, exercise duration, sleep duration on workdays, and sleep duration on non-workdays as covariates, as identified by the DAG.

Full matching was applied using the MatchIt package in R to improve covariate balance while retaining the maximum number of observations. Covariate balance before and after matching was assessed using standardized mean differences (SMD), with SMD < 0.10 indicating adequate balance. Balance diagnostics were additionally visualized using Love plots.

In the matched sample, logistic regression models were used to examine the association between mental health service utilization and diabetes–hypertension comorbidity. Matching weights and subclassification (matched_id) were incorporated into the regression model to account for the matched design. Results were reported as odds ratios (ORs) with 95% confidence intervals (CIs), and forest plots were used to display effect estimates. Sensitivity analyses were conducted by sequentially excluding key covariate groups to assess the robustness of the estimated associations. Subgroup and interaction analyses were further conducted to explore potential heterogeneity in the association between mental health service utilization and DM–HTN comorbidity. Interaction terms between mental health service utilization and key variables, including age, gender, income status, and depression status, were incorporated into logistic regression models. Predicted probabilities were subsequently calculated to illustrate differences in DM–HTN comorbidity risk across relevant subgroups.

All analyses were conducted using R version 4.4.2. The dagitty and ggdag packages were used for DAG construction. Propensity score matching was implemented using MatchIt, and covariate balance was evaluated using the cobalt package. Logistic regression analyses were performed using the stats package. ROC curves were generated using the pROC package, and model performance was assessed using AIC, BIC, and calibration curves. Multicollinearity was evaluated using the generalized variance inflation factor (GVIF) from the car package.

## Results

A total of 218 participants (16.7%) had comorbid diabetes and hypertension, while 1,089 (83.3%) did not. The proportion of males was significantly higher in the comorbidity group compared with the non-comorbidity group (50.9% vs. 42.0%, *p* = 0.015). Age distribution differed markedly between groups (*p* < 0.001). Participants aged 65–79 years accounted for the largest proportion in the comorbidity group (41.3% vs. 25.8%), and those aged ≥80 years were also more prevalent (8.7% vs. 5.8%). In contrast, younger adults aged 18–44 years were substantially underrepresented in the comorbidity group (13.8% vs. 37.2%).

Marital status was significantly associated with comorbidity status (*p* < 0.001). Individuals who were married and living with a partner comprised a higher proportion of the comorbidity group (36.7% vs. 23.5%), whereas those married but not living with a partner were less common (13.8% vs. 20.7%). The proportion of never-married individuals was lower in the comorbidity group (49.5% vs. 55.8%).

Educational attainment also differed significantly (*p* = 0.006). Participants with a bachelor’s degree or above were less represented in the comorbidity group (28.4% vs. 38.2%), while those with lower educational levels (up to grade 11) were proportionally more common. A higher proportion of participants in the comorbidity group were living in poverty (22.5% vs. 15.3%, *p* = 0.010).

Sleep duration showed significant group differences for both weekdays (*p* = 0.004) and non-working days (*p* = 0.001). Short sleep duration (≤4 h) was more prevalent among individuals with comorbidity on both weekdays (5.5% vs. 1.7%) and non-working days (4.6% vs. 1.8%). On non-working days, a smaller proportion of the comorbidity group reported ≥8 h of sleep (59.2% vs. 70.5%).

There were also significant differences in the exercise patterns. Individuals with comorbidities had a higher proportion of exercising more than 30 times per month (56.9% vs. 42.8%, *p* = 0.003). Additionally, they also had a higher proportion of no exercise duration (34.4% vs. 19.0%, *p* < 0.001).

Regarding health-related behaviors, smoking (52.3% vs. 39.4%, *p* < 0.001) and alcohol consumption (45.9% vs. 33.1%, *p* < 0.001) were both significantly more prevalent among participants with DM–HTN comorbidity. Depression was also more common in the comorbidity group (11.9% vs. 7.8%, *p* = 0.046). In contrast, utilization of mental health services was lower among individuals with comorbidity (11.9% vs. 17.5%, *p* = 0.042) (Table 1).

**Table 1:** Sociodemographic characteristics by DM-HTN comorbidity.

Variable		Yes (*n* = 218)	No (*n* = 1,089)	*p*
Gender	Male	111 (50.9)	457 (42.0)	0.015
	Female	107 (49.1)	632 (58.0)	
Age	18–44	30 (13.8)	405 (37.2)	<0.001
	45–64	79 (36.2)	340 (31.2)	
	65–79	90 (41.3)	281 (25.8)	
	≥80	19 (8.7)	63 (5.8)	
Marriage	Never married	108 (49.5)	608 (55.8)	<0.001
	Married and partner living	80 (36.7)	256 (23.5)	
	Married not living with partner	30 (13.8)	225 (20.7)	
Education	Up to grade 9	13 (6.0)	39 (3.6)	0.006
	Grades 9–11	27 (12.4)	76 (7.0)	
	Graduate from high school	54 (24.8)	242 (22.2)	
	Three-year college	62 (28.4)	316 (29.0)	
	Bachelor’s degree and above	62 (28.4)	416 (38.2)	
Income	Poverty	49 (22.5)	167 (15.3)	0.010
	Not poor	169 (77.5)	922 (84.7)	
Sleep duration on weekdays	≤4 h	12 (5.5)	19 (1.7)	0.004
	4.5–7.5 h	94 (43.1)	479 (44.0)	
	≥8 h	112 (51.4)	591 (54.3)	
Sleep duration on non-working days	≤4 h	10 (4.6)	20 (1.8)	0.001
	4.5–7.5 h	79 (36.2)	301 (27.6)	
	≥8 h	129 (59.2)	768 (70.5)	
Exercise frequency	1 day	10 (4.6)	89 (8.2)	0.003
	2–7 days	25 (11.5)	141 (12.9)	
	8–14 days	21 (9.6)	131 (12.0)	
	15–30 days	38 (17.4)	262 (24.1)	
	>30 days	124 (56.9)	466 (42.8)	
Exercise duration	0	75 (34.4)	207 (19.0)	<0.001
	≤0.5 h	70 (32.1)	363 (33.3)	
	0.5–1 h	52 (23.9)	337 (30.9)	
	1–2 h	14 (6.4)	147 (13.5)	
	≥2 h	7 (3.2)	35 (3.2)	
Smoking	YES	114 (52.3)	429 (39.4)	<0.001
Drinking	YES	100 (45.9)	361 (33.1)	<0.001
Depression	YES	26 (11.9)	85 (7.8)	0.046
Mental health services	YES	26 (11.9)	191 (17.5)	0.042

All of the above were tested by chi-square test.

Previous research has demonstrated that demographic variables such as age, gender have direct effects on the comorbidity of diabetes and hypertension. Marital status may influence smoking, alcohol consumption, depression, and access to mental health services, thereby exerting an indirect effect on Diabetes-Hypertension (DM-HTN) comorbidity. Educational attainment has been shown to affect income level. Among socioeconomic factors, income may impact exercise frequency, smoking, alcohol consumption, and depression, all of which can indirectly influence DM-HTN comorbidity. Regarding health behavior variables, exercise frequency affects the duration of physical activity, and both have direct effects on DM-HTN comorbidity. Sleep duration on workdays influences sleep on non-workdays, and workday sleep is also directly associated with DM-HTN comorbidity. Depression may influence both smoking and drinking behaviors, and all three are directly linked to DM-HTN comorbidity.

**Figure 1:**
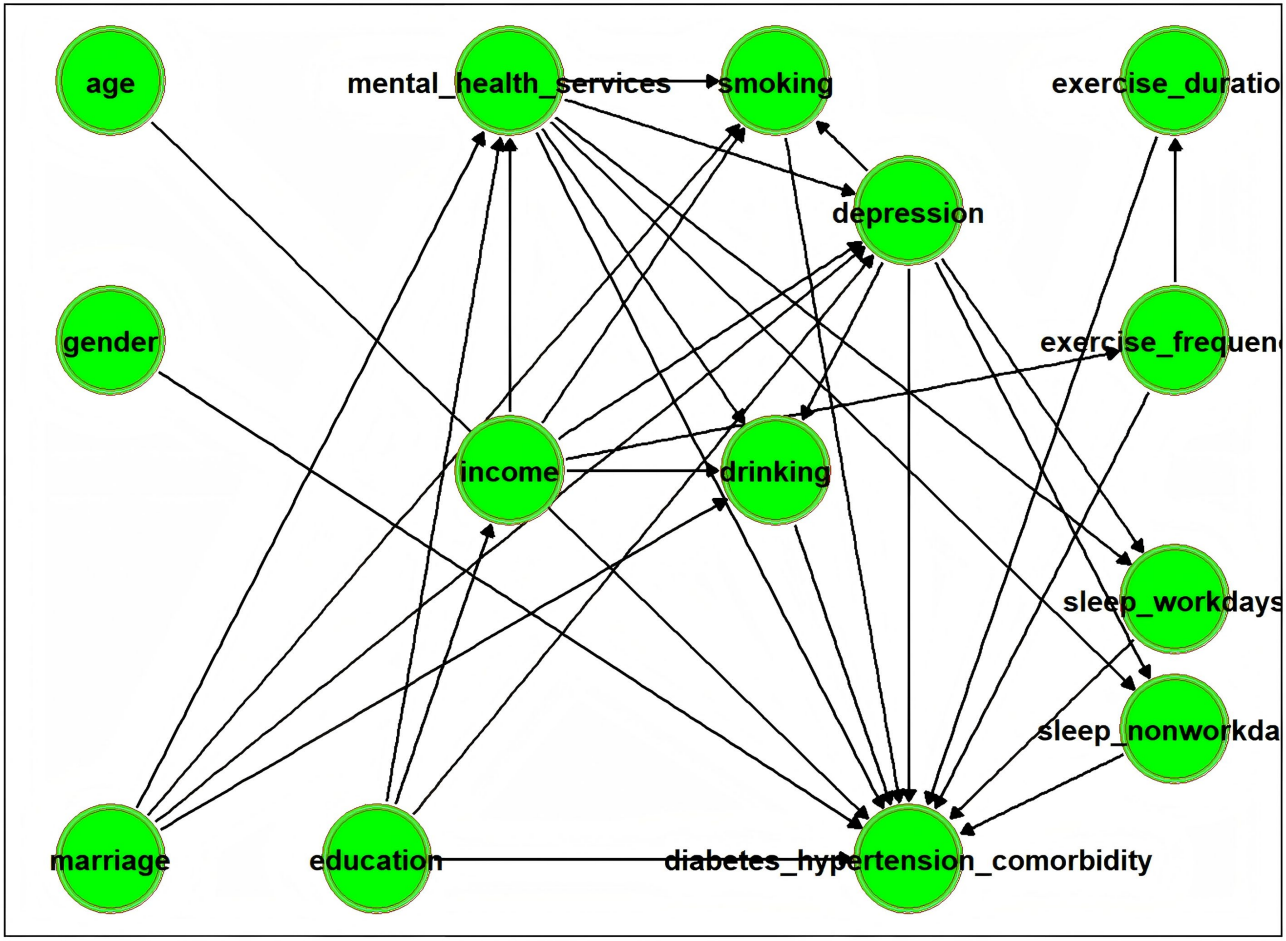
DAG of comorbidity between diabetes and hypertension.

**Figure 2:**
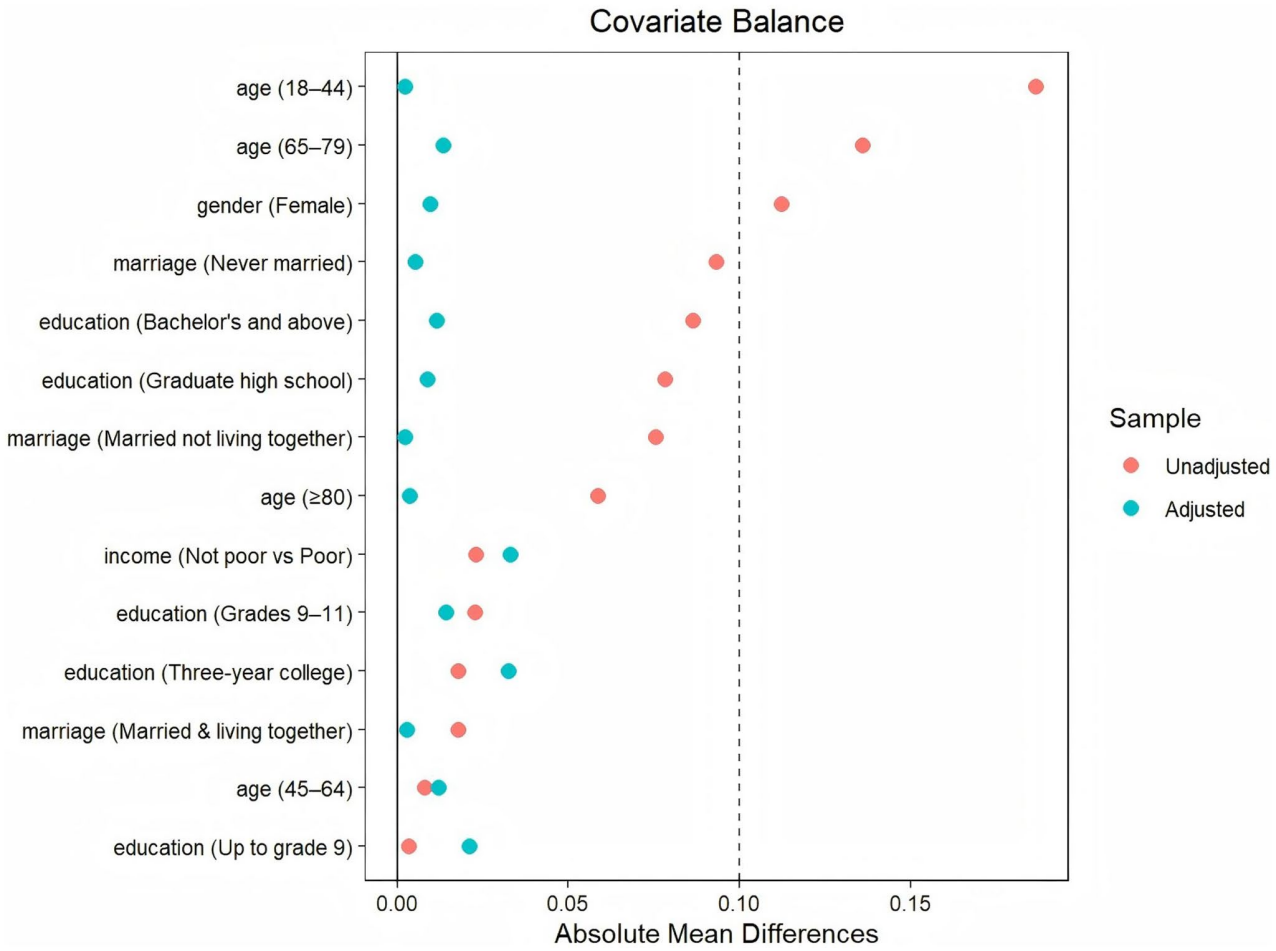
Covariate balance assessment after PSM.

Based on prior knowledge and existing literature, we constructed a Directed Acyclic Graph (DAG) to represent these assumed causal relationships (Figure 1) (17–23). According to the DAG structure, we identified key covariates-including age, gender, income, educational level, and marital status-for adjustment through propensity score matching. The standardized mean difference (SMD) after matching was controlled below 0.1, indicating an adequate balance of covariates and good matching quality for subsequent analysis (Figures 1, 2).

n the multivariable logistic regression, mental health service utilization was not significantly associated with DM–HTN comorbidity after adjusting for sociodemographic factors in Model 1 (*β* = −0.228, SE = 0.236, *Z* = −0.967, *p* = 0.333). After further adjustment for depression, health behaviors, and sleep-related variables in Model 2, the association became statistically significant (*β* = −0.537, SE = 0.237, *Z* = −2.266, *p* = 0.023) (Table 2).

**Table 2:** Association between mental health service utilization and risk of DM–HTN comorbidity: multivariable logistic regression models.

Model	Variables	Estimate (mental health services)	Std. error (mental health services)	*z-*value (mental health services)	*p-*value (mental health services)
Model 1	Mental health services, age, gender, income, education, marriage	−0.228	0.236	−0.967	0.333
Model 2	Mental health services, depression, smoking, drinking, exercise frequency, exercise duration, sleep duration on weekdays, sleep duration on non-working days	−0.537	0.237	−2.266	0.023

**Figure 3:**
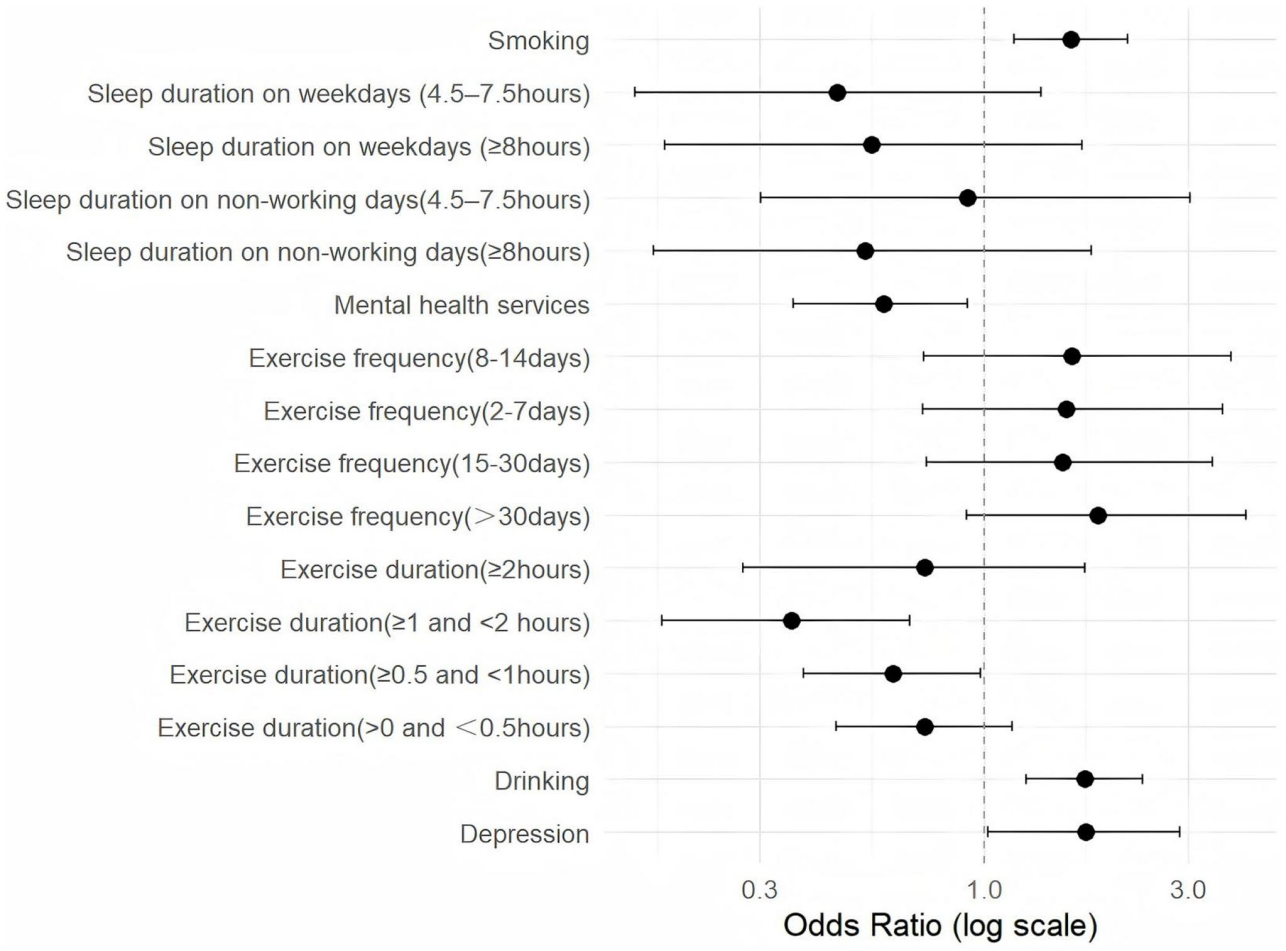
Forest plot of factors influencing diabetes and hypertension comorbidity.

Mental health service utilization was significantly associated with a reduced likelihood of DM-HTN comorbidity (*OR* = 0.584, *95% CI*: 0.360–0.915, *p* = 0.024). Depression was significantly associated with increased odds of DM-HTN comorbidity (*OR* = 1.730, *95% CI*: 1.024–2.862, *p* = 0.035). Similarly, smoking (*OR* = 1.600, *95% CI*: 1.177–2.171, *p* = 0.003) and alcohol consumption (*OR* = 1.720, *95% CI*: 1.253–2.350, *p* < 0.001) were independently associated with higher risk. Regarding physical activity frequency, none of the exercise frequency categories reached statistical significance compared with the reference group (everyday). Compared with no exercise, exercising 0.5 to <1 h per session (*OR* = 0.614, *95% CI*: 0.379–0.984, *p* = 0.045) and exercising 1 to <2 h per session (*OR* = 0.355, *95% CI*: 0.177–0.671, *p* = 0.002) were associated with lower odds of comorbidity. Shorter durations (>0 to <0.5 h) and ≥2 h were not statistically significant. Sleep duration on weekdays and non-working days was not significantly associated with DM-HTN comorbidity (Table 3 and Figure 3).

**Table 3:** Association between mental health services and risk of comorbid diabetes and hypertension after PSM.

Variable	Estimate	Std. error	*Z* value	*p-*value	*OR*	*95% CI*
Intercept	−1.050	0.585	−1.800	0.072	0.350	0.108 ~ 1.079
Mental health services	−0.537	0.237	−2.270	0.024	0.584	0.360 ~ 0.915
Depression	0.550	0.261	2.100	0.035	1.730	1.024 ~ 2.862
Smoking	0.469	0.156	3.010	0.003	1.600	1.177 ~ 2.171
Drinking	0.540	0.160	3.370	<0.001	1.720	1.253 ~ 2.350
Exercise frequency (2–7 days)	0.442	0.408	1.080	0.278	1.560	0.723 ~ 3.783
Exercise frequency (8–14 days)	0.473	0.418	1.130	0.258	1.600	0.835 ~ 1.958
Exercise frequency (15–30 days)	0.420	0.388	1.080	0.279	1.520	0.736 ~ 3.420
Exercise frequency (>30 days)	0.614	0.380	1.620	0.106	1.850	0.911 ~ 4.097
Exercise duration (>0 and <0.5 h)	−0.317	0.240	−1.320	0.187	0.728	0.453 ~ 1.163
Exercise duration (≥0.5 and <1 h)	−0.488	0.243	−2.010	0.045	0.614	0.3793 ~ 0.984
Exercise duration (≥1 and <2 h)	−1.040	0.338	−3.070	0.002	0.355	0.177 ~ 0.671
Exercise duration (≥2 h)	−0.316	0.463	−0.683	0.495	0.729	0.274 ~ 1.719
Sleep duration on weekdays (4.5–7.5 h)	−0.790	0.550	−1.440	0.151	0.454	0.154 ~ 1.361
Sleep duration on weekdays (≥8 h)	−0.604	0.566	−1.070	0.286	0.547	0.180 ~ 1.695
Sleep duration on non-working days (4.5–7.5 h)	−0.090	0.584	−0.153	0.878	0.914	0.301 ~ 3.035
Sleep duration on non-working days (≥8 h)	−0.638	0.595	−1.070	0.284	0.528	0.170 ~ 1.786

The analysis was conducted on the propensity score matched sample, with matching variables including education, income, marital status, gender, and age, using full matching method implemented via the MatchIt package.

Sensitivity analyses showed that the association between mental health service utilization and DM–HTN comorbidity remained generally stable across multiple model specifications. When depression was excluded from adjustment, the association was attenuated and became marginally non-significant (OR = 0.638, 95% CI: 0.406–1.005). In contrast, excluding smoking and alcohol consumption slightly strengthened the association (OR = 0.574, 95% CI: 0.362–0.910). Removing physical activity and sleep-related variables produced negligible changes in effect estimates. Importantly, even in the crude model without covariate adjustment, mental health service utilization remained significantly associated with lower odds of comorbidity (OR = 0.637, 95% CI: 0.411–0.987), indicating robustness of the observed association (Supplementary Table S1).

In the subgroup and interaction analysis, no statistically significant interactions were observed between mental health service utilization and age groups (45–64 years: OR = 0.64, *p* = 0.428; 65–79 years: OR = 0.427, *p* = 0.208; ≥80 years: OR = 2.19, *p* = 0.564), gender (female: OR = 0.853, *p* = 0.733), or income status (not poor: OR = 1.56, *p* = 0.402). However, a statistically significant interaction was observed between mental health service utilization and depression status (OR = 0.587, 95% CI: 0.359–0.960, *p* = 0.033) (Table 4).

**Table 4:** Interaction effects between mental health service utilization and key sociodemographic and psychological factors on DM–HTN comorbidity.

Variable	Estimate	Std. error	*Z* value	*p-*value	*OR*	*95% CI*
Intercept	−1.98	0.367	−5.400	<0.001	0.138	0.065 ~ 0.276
Mental health services (YES) * age (45–64)	−0.446	0.563	−0.792	0.428	0.640	0.213 ~ 1.980
Mental health services (YES) * age (65–79)	−0.852	0.677	−1.260	0.208	0.427	0.107 ~ 1.590
Mental health services (YES) * age (≥80)	0.783	1.360	0.577	0.564	2.190	0.087 ~ 29.500
Mental health services (YES) * gender (female)	−0.159	0.467	−0.341	0.733	0.853	0.341 ~ 2.160
Mental health services (YES) * income (not poor)	0.446	0.532	0.838	0.402	1.560	0.539 ~ 0.960
Mental health services (YES) * depression (YES)	−0.533	0.251	−2.124	0.033	0.587	0.359 ~ 0.960

Predicted probability analysis showed a consistent protective association of mental health service utilization across depression strata. Among individuals without depression, the predicted probability of DM–HTN comorbidity was 0.0707 for non-users and 0.0520 for users of mental health services, corresponding to an absolute risk reduction of 1.87%. Among individuals with depression, the predicted probabilities were 0.1281 and 0.0978, respectively, indicating a larger absolute risk reduction of 3.03% (Table 5).

**Table 5:** Predicted probabilities of DM–HTN comorbidity by mental health service utilization and depression status.

Depression	Mental health services	Predicted probability
No	No	0.0707
No	Yes	0.0520
Yes	No	0.1281
Yes	Yes	0.0978

The model achieved an accuracy of 0.83, suggesting good discriminative performance in distinguishing individuals with versus without comorbid diabetes and hypertension (Supplementary Figure 1). The Akaike Information Criterion (AIC) and Bayesian Information Criterion (BIC) were 1136.844 and 1224.827, respectively, indicating an acceptable overall model fit. The calibration curve of the logistic regression model showed close agreement between estimated and observed probabilities, supporting good calibration. To assess the model’s robustness and internal stability, we conducted 200 bootstrap resampling iterations. The resulting mean absolute error was 0.0009, the mean squared error was 0.025, and the 90th percentile error was 0.057, indicating stable performance (Supplementary Figure 2). Multicollinearity among independent variables was evaluated using the Generalized Variance Inflation Factor (GVIF); all GVIF^[1/(2*Df)] values were below 1.3, suggesting no meaningful multicollinearity among predictors.

## Discussion

This study was based on a multistage random sampling design and a community-based cross-sectional survey conducted in 10 communities in Urumqi. A Directed Acyclic Graph (DAG) was used to identify potential confounders, and Propensity Score Matching (PSM) was applied to reduce confounding bias. The findings indicated that mental health service utilization was associated with a lower likelihood of DM–HTN comorbidity, whereas depression, smoking, and alcohol consumption were associated with a higher likelihood of comorbidity. In addition, moderate exercise duration was associated with reduced odds of DM–HTN comorbidity.

Multiple studies have shown that mental health services play a vital role in chronic disease intervention, especially in regulating psychological stress and improving health behaviors. Cognitive Behavioral Therapy (CBT) and psychological counseling have been proven to help reduce the overactivation of the HPA axis and sympathetic nervous system by improving coping strategies and emotional regulation, thereby contributing to the stabilization of blood glucose and blood pressure levels (24, 25). In addition, psychological interventions can improve sleep quality and promote willingness to exercise, making them an important part of chronic disease management (26). Notably, the association between mental health service utilization and DM–HTN comorbidity was not statistically significant after adjustment for sociodemographic confounders alone (Model 1). However, after further incorporating behavioral and psychological variables, including depression, smoking, alcohol consumption, physical activity, and sleep-related factors, mental health service utilization became significantly associated with a lower likelihood of DM–HTN comorbidity (Model 2). This pattern suggests that behavioral and psychological factors may play an important role in the pathway linking mental health service utilization to chronic disease outcomes. A related study investigating the association between the use of mental health services and the risk of diabetes did not find a significant effect, possibly due to inadequate confounding control, thus underestimating the effect of the services (27). In contrast, this study identified confounders based on a DAG and controlled for bias using PSM, which enhances the causal explanatory power of the results from both methodological rigor and confounding control perspectives.

The phased modelling strategy adopted in this study allowed a clearer distinction between potential confounding factors and behavioral or psychological variables that may lie along the causal pathway. Sociodemographic characteristics were treated as pre-exposure confounders, whereas depression, smoking, drinking, physical activity, and sleep-related variables were considered variables that may lie on the pathway linking mental health service utilization and chronic disease outcomes. Although mediation effects cannot be formally established using cross-sectional data, the findings provide preliminary evidence that mental health services may exert their protective association partly through improving psychological well-being and promoting healthier behaviors.

This study found that depression was significantly associated with an increased risk of comorbid diabetes and hypertension, supporting the bidirectional relationship between chronic physical conditions and mental health disorders. On one hand, depression can activate the HPA axis and sympathetic nervous system over the long term, leading to insulin resistance, vascular constriction, and immune dysregulation, thereby exacerbating chronic disease risk at the physiological level (28). On the other hand, patients with diabetes and hypertension are commonly accompanied by experience helplessness and anxiety, forming a negative feedback loop (29).

Beyond its independent association with DM–HTN comorbidity, depression also modified the relationship between mental health service utilization and chronic disease outcomes. Interaction analysis demonstrated a significant interaction between mental health service utilization and depression status. No significant interactions were observed for age, gender, or income, indicating that depression was the primary factor modifying the association between mental health service utilization and DM–HTN comorbidity. Predicted probability analysis further showed that the absolute reduction in DM–HTN comorbidity associated with mental health service utilization was larger among individuals with depression (3.03%) than among those without depression (1.87%). These findings suggest that individuals experiencing depressive symptoms appeared to show a stronger association between mental health service utilization and lower DM–HTN comorbidity risk, potentially because such interventions directly target emotional regulation, treatment adherence, and health-related self-management behaviors. From a public health perspective, prioritizing mental health service access among psychologically vulnerable populations may therefore yield greater health benefits. These findings highlight the potential value of targeting mental health service resources toward individuals with depressive symptoms when designing chronic disease prevention and management strategies.

This study also demonstrated that smoking and alcohol consumption were significantly associated with an increased risk of comorbid diabetes and hypertension. Research has shown that smoking can cause endothelial dysfunction, chronic inflammation, and decreased insulin sensitivity, accelerating the progression of diabetes and hypertension (30). Frequent alcohol consumption can damage liver metabolism, promote weight gain, and cause blood glucose fluctuations, which further trigger Diabetes-Hypertension (DM-HTN) comorbidity (31). Although some studies have suggested that moderate drinking may benefit the cardiovascular system (32), our findings indicate that alcohol consumption shows an overall negative effect in the general population, possibly due to high frequency and volume of drinking. This suggests that public policies and clinical interventions should pay attention to the specific patterns of alcohol use.

To complement the conventional regression analysis, a machine learning approach based on random forest modelling was conducted to explore potential nonlinear relationships and rank predictor importance. The model achieved a moderate discriminative ability (AUC = 0.661 in the testing dataset), indicating reasonable predictive performance. Variable importance analysis identified age, exercise duration, educational attainment, and exercise frequency as the strongest predictors of DM–HTN comorbidity. Mental health service utilization showed relatively lower predictive importance. However, predictor importance reflects contribution to outcome prediction rather than causal relevance. Therefore, while mental health service utilization may explain a smaller proportion of overall population-level variation, it remained significantly associated with a lower likelihood of DM–HTN comorbidity in the multivariable regression analysis after adjustment for confounding factors.

This study found that moderate-duration physical activity may be associated with a reduction in the DM-HTN comorbidity, whereas insufficient exercise may not reach the intensity or duration required to induce meaningful physiological adaptations. Regular moderate exercise improves insulin sensitivity, enhances endothelial function, reduces systemic inflammation, and promotes blood pressure regulation. In addition, physical activity may exert indirect protective effects through psychological pathways, including stress reduction and mood improvement, which are particularly relevant given the observed association between depression and DM-HTN comorbidity (33).

Despite the relatively robust conclusions achieved through Directed Acyclic Graphs (DAGs) and Propensity Score Matching (PSM) methods in this study, some limitations remain to be addressed in future research. Although PSM is advantageous in reducing the influence of confounding variables, it only controls for observable factors and cannot adjust for unobservable variables such as genetic predisposition or social support (34). Second, all information on mental health service utilization, depression status, smoking, alcohol consumption, physical activity, and sleep duration was self-reported and may therefore be subject to recall bias and social desirability bias. Third, participants were recruited from communities in Urumqi, Xinjiang, and the findings may not be directly generalizable to populations with different demographic compositions, cultural contexts, or healthcare systems. Moreover, propensity score matching may reduce the effective sample size and consequently affect statistical power when unmatched participants are excluded from the analysis, as discussed by Stuart (36). In addition, simulation studies have shown that propensity score methods do not necessarily outperform conventional logistic regression approaches in all situations and may exhibit lower statistical efficiency under certain conditions (35). As reported by Halstead et al. (37), the prevalence and influencing factors of multimorbidity vary significantly across populations with or without mental illnesses in different regional and healthcare contexts. In addition, retrospective cohort studies adopting PSM design have confirmed that regional population heterogeneity greatly affects the extrapolation of research conclusions (38). Future studies could incorporate instrumental variable methods or Bayesian network analysis to further enhance the accuracy of causal inference. Since this study is based on cross-sectional data, it is difficult to directly infer long-term causal relationships. Prospective cohort studies or randomized controlled trials (RCTs) are needed to further verify the impact of mental health services on diabetes and hypertension (DM-HTN) comorbidity. In addition, sensitivity analyses for unmeasured confounding were not performed, and residual confounding from unobserved factors cannot be excluded. In addition, this study did not distinguish between different types of mental health interventions (e.g., psychological counseling, cognitive behavioral therapy, medication), and future research could explore the differential effects of these interventions to provide more refined evidence for precision health management.

In conclusion, this study employed Directed Acyclic Graphs (DAGs) and Propensity Score Matching (PSM) to examine the associations between mental health services, depression, smoking, drinking, physical activity, and sleep duration with the comorbidity of diabetes and hypertension (DM-HTN). The findings indicated that receiving mental health services was associated with a lower likelihood of DM-HTN comorbidity. This association appeared to be stronger among individuals with depression, suggesting that the association between mental health service utilization and DM–HTN comorbidity may be more pronounced among psychologically vulnerable populations. Conversely, depression, smoking, and alcohol consumption were associated with an increased risk, highlighting the need for integrated mental health and behavioral interventions in chronic disease management.

Moreover, physical activity was negatively associated with DM-HTN comorbidity, underscoring the importance of lifestyle modification as a preventive strategy. While these results provide valuable insights, the cross-sectional nature of the data precludes causal inference. Therefore, future longitudinal studies are warranted to further investigate these relationships. Policymakers should consider enhancing the accessibility and effectiveness of mental health services, particularly among individuals experiencing depressive symptoms, while simultaneously promoting healthy lifestyle behaviors to reduce the burden of chronic disease comorbidities and improve population health outcomes.

## Data Availability

The raw data supporting the conclusions of this article will be made available by the authors, without undue reservation.
