# Supplementary material for: Association between mental health service utilization and diabetes-hypertension comorbidity: a community-based study
Source: Front Public Health. 2026 Jul 3;14:1835225. doi: 10.3389/fpubh.2026.1835225 (PMC13375783; doi:10.3389/fpubh.2026.1835225)
Supplement: Supplementary file 1 [file Table_1.DOC]

**Supplementary Table S1.** Sensitivity analyses of the association between mental health service utilization and diabetes–hypertension comorbidity.

| Model | Covariates removed | *OR* | *95% CI* | *P value* |
| --- | --- | --- | --- | --- |
| A | None | 0.584 | 0.367–0.930 | 0.024 |
| B | Depression | 0.638 | 0.406–1.005 | 0.053 |
| C | Smoking, alcohol use | 0.574 | 0.362–0.910 | 0.018 |
| D | Exercise frequency, exercise duration | 0.581 | 0.366–0.921 | 0.021 |
| E | Sleep duration (workdays and non-workdays) | 0.589 | 0.372–0.932 | 0.024 |
| F | All behavioral/psychological covariates | 0.637 | 0.411–0.987 | 0.044 |

**Note.** Across all sensitivity analyses, the direction and magnitude of the association remained broadly consistent (OR range: 0.574–0.638), suggesting that the observed association was not driven by any single psychological or behavioral covariate. All models were fitted using binary logistic regression on the full analytic sample (N = 1,307). Categorical factor coding was used for exercise frequency (reference: 1 day/month), exercise duration (reference: no exercise), and sleep duration on workdays and non-workdays (reference: ≤4 hours). MHS = mental health services; OR = odds ratio; CI = confidence interval; DM–HTN = diabetes–hypertension comorbidity.

**
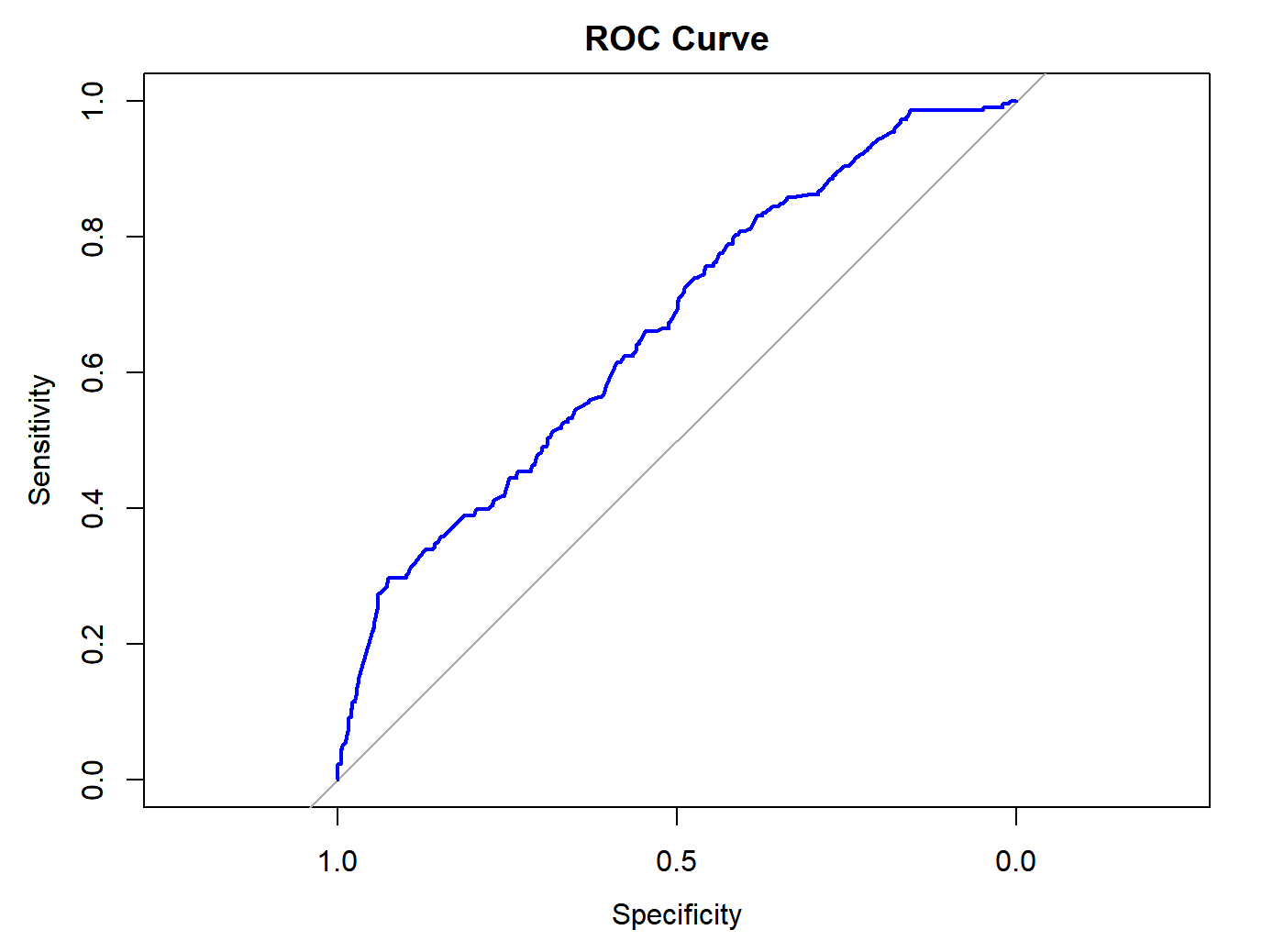

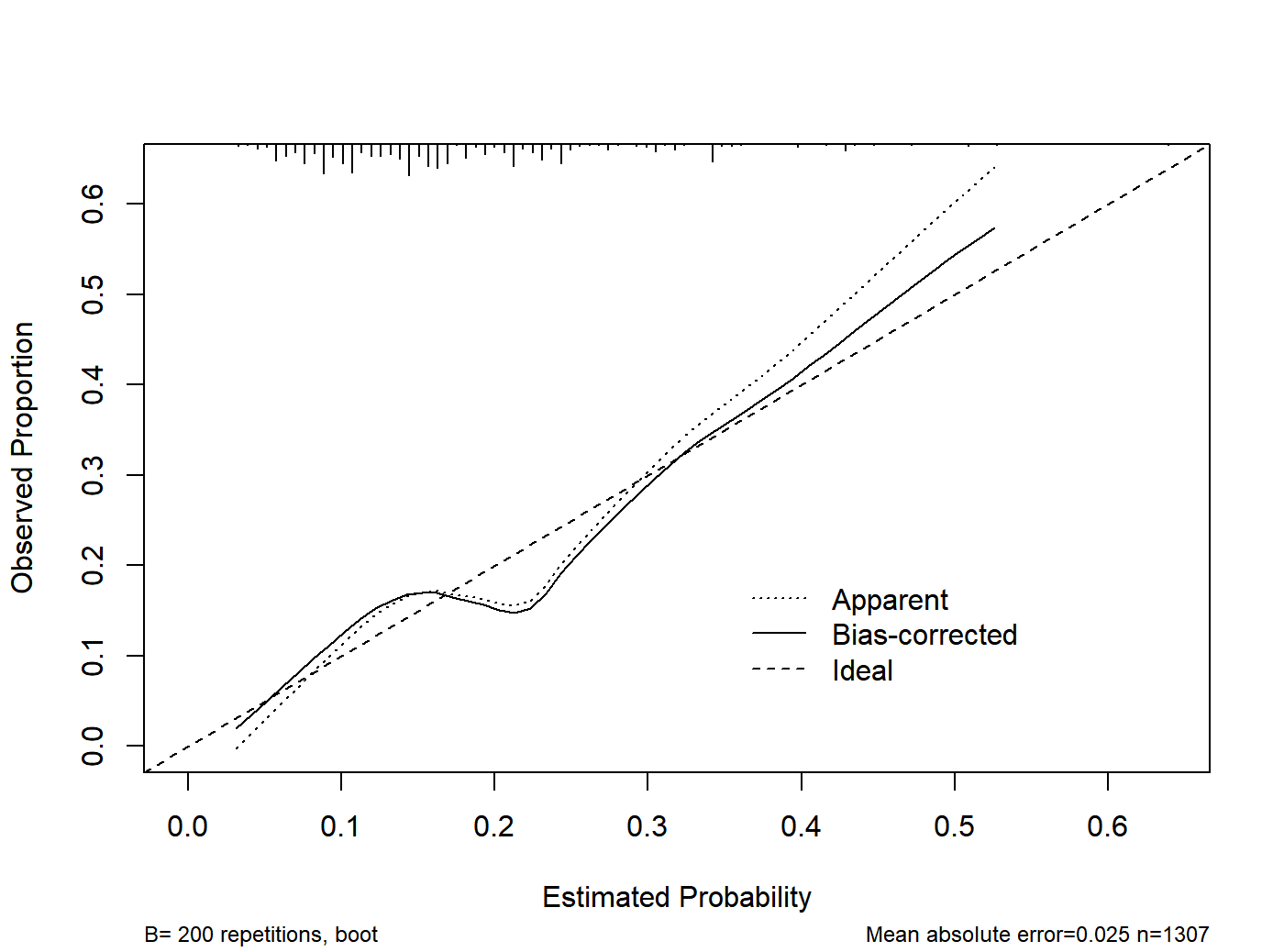
**

**Supplementary Figure 1. ROC Curve Supplementary Figure 2. Calibration curve**
